# Expression and Localization of an Hsp70 Protein in the Microsporidian *Encephalitozoon cuniculi*

**DOI:** 10.1155/2010/523654

**Published:** 2010-07-27

**Authors:** Carrie E. Jolly, Cory A. Leonard, J. Russell Hayman

**Affiliations:** Department of Microbiology, James H. Quillen College of Medicine, East Tennessee State University, Box 70579, Johnson City, TN 37614, USA

## Abstract

Microsporidia spore surface proteins are an important, under investigated aspect of spore/host cell attachment and infection. For comparison analysis of surface proteins, we required an antibody control specific for an intracellular protein. An endoplasmic reticulum-associated heat shock protein 70 family member (Hsp70; ECU02_0100; “C1”) was chosen for further analysis. DNA encoding the C1 hsp70 was amplified, cloned and used to heterologously express the C1 Hsp70 protein, and specific antiserum was generated. Two-dimensional Western blotting analysis showed that the purified antibodies were monospecific. Immunoelectron microscopy of developing and mature *E. cuniculi* spores revealed that the protein localized to internal structures and not to the spore surface. In spore adherence inhibition assays, the anti-C1 antibodies did not inhibit spore adherence to host cell surfaces, whereas antibodies to a known surface adhesin (EnP1) did so. In future studies, the antibodies to the ‘C1' Hsp70 will be used to delineate spore surface protein expression.

## 1. Introduction

Microsporidia are spore-forming, obligate intracellular divergent fungi with an extensive host range that includes most vertebrates and invertebrates. Although the first species of microsporidia was described over 150 years ago, microsporidiosis was rarely diagnosed in humans prior to the AIDS pandemic. Today, microsporidia are recognized as opportunistic pathogens of humans [1]. Most microsporidia infections in humans are thought to arise via the fecal-oral route. Ingestion of the environmentally stable spores leads to primary infection in the small intestine where replication of the organisms results in destruction of the epithelium. Therefore, the most common clinical manifestations of microsporidiosis are self-limiting diarrhea in immunocompetent individuals and persistent diarrhea perhaps leading to a wasting syndrome in the immunocompromised [2]. 

All microsporidia possess a unique invasion apparatus known as the polar tube or polar filament, which must be discharged in order to infect the host cell. Upon extrusion, the polar tube penetrates the host cell plasma membrane and allows the passage of infectious sporoplasm from the spore through the hollow polar tube into the host cell cytoplasm where replication occurs. We hypothesize that infection of the host cell is facilitated by adherence of the microsporidia spore to the host cell surface prior to or during the activation process. Our previous studies have demonstrated that microsporidia spores of the genus *Encephalitozoon* adhere to the host cell surface *in vitro* through at least one mechanism involving host cell glycosaminoglycans [3]. *In vitro* spore adherence and host cell infectivity assays demonstrate that addition of exogenous sulfated glycosaminoglycans to the culture medium results in decreased spore adherence and decreased number of infected host cells. Our studies indicate a direct association between microsporidia adherence to the host cell surface and infectivity.

To understand the mechanism of microsporidia adherence, we have turned our attention to identifying possible ligands on the spore surface. Putative proteins with recognizable adhesion domains are identified by searching the *Encephalitozoon cuniculi* genome database with adhesion/attachment motifs. Identified proteins are heterologously expressed, purified, and used for antibody production. We are using previously developed assays to evaluate the recombinant proteins and their corresponding antibodies as potential inhibitors of spore adherence and/or host cell infectivity. For comparison purposes, we require a microsporidia protein that is not located on the spore surface and does not inhibit spore adherence or infectivity. Because heat shock proteins (Hsps) are typically found in the cytosol, ER, and mitochondria of a cell [4], we chose to examine the Hsp70-related proteins from *E. cuniculi* as potential candidates. In this study, we demonstrate by transmission immunoelectron microscopy that the Hsp70-related protein (ECU02_0100) is located in internal structures of the spore. We also show that antibodies against the recombinant Hsp70-related protein C1 do not significantly inhibit spore adherence or host cell infection *in vitro*. Recombinant Hsp70 protein and the antibodies against this protein will be used for comparison purposes in our quest to identify possible microsporidia spore ligands that function during adherence.

## 2. Materials and Methods

### 2.1. Microsporidia and Host Cell Cultivation

 African green monkey kidney cells (Vero; ATCC CCL-81) and rabbit kidney cells (RK-13; ATCC CCL-37) were used for the cultivation of microsporidia spores. Adherent cells were maintained in Dulbecco's modified Eagle's medium (BioWhittaker, Walkersville, MD) supplemented with L-glutamine (2 mM), penicillin (100 U/mL), streptomycin (100 *μ*g/mL), amphotericin B (0.25 *μ*m/mL), and 2% fetal bovine serum (BioWhittaker) in 5% CO_2_ at 37°C. Microsporidia spore propagation and purification were performed as previously described in [5].

### 2.2. Recombinant Protein Expression and Antiserum Production

 The gene encoding the “C1” Hsp70-related protein (ECU02_0100) was PCR amplified from *E. cuniculi* genomic DNA using the following primers; 5′-GGAATTCATGAACAAGGGTATGCTAG-3′ and 5′-ACTCGAGGAGTTCTTCTCTCCCTATTTC-3′. The amplicon was cloned into the pET21a vector (EMD Biosciences, Inc., Madison, WI) using restriction endonucleases and ligation. Following transformation into *Escherichia coli* Rosetta Gami cells (EMD Biosciences) and induction with IPTG (isopropyl-*β*-D-thiogalactopyranoside), the bacterial harvest was sonicated in PBS containing 5% SDS and 2%  *β*-mercaptoethanol. SDS-PAGE gels were Coomassie stained, and Western analysis was performed using a histidine-tag-specific antibody (Sigma-Aldrich, St. Louis, MO) to confirm recombinant protein expression.

For purification, the bacterial pellet was sonicated in column chromatography binding buffer containing 8 M urea and 20 mM imidazole in 1X phosphate buffered saline. The supernatant was applied to an equilibrated nickel affinity column (GE Biosciences, Piscataway, JH). Following washes with binding buffer, the recombinant protein was eluted with 1X phosphate buffered saline containing 8 M urea and 300 mM imidazole. Fractions containing recombinant protein were combined and dialyzed against 10 mM Tris buffer (pH 7.4) with 0.5 mM EDTA. The resulting dialysate was centrifuged, and the supernatant containing the protein was pooled and stored at −20°C for further use. 

For antiserum production, naïve rabbits were immunized with the recombinant C1 Hsp70-related protein using a 56-day immunization protocol conducted at a commercial facility (Proteintech Group, Inc., Chicago, IL). Antibodies from both pre- and postimmunized rabbits were purified from serum using protein A/G affinity chromatography according to the manufacturer's recommendations (Thermo Fisher Scientific, Rockford, IL).

### 2.3. Spore Adherence Assays

 Microsporidia spore adherence assays were performed as previously described in [3]. RK13 cells were seeded onto round glass coverslips in 12-well plates and grown to confluence. To test adherence, purified diluted anti-C1 antibodies (1 mg/mL) or anti-EnP1 antibodies (1 mg/mL) [6] were incubated with *E. cuniculi* spores in medium on RK13 host cells for 4-hours on ice. The unbound spores were removed by washing with PBS, and the bound spores were quantified by immunofluorescence as described in [6]. The results are expressed as the percentage of adherent spores relative to control samples. Statistical significance was determined using the Student's *t* test.

### 2.4. SDS-PAGE Analysis and Western Blotting

 One-dimensional sodium dodecyl sulfate polyacrylamide gel electrophoresis (1D-SDS-PAGE) was performed with purified spore protein as previously described in [6]. For two-dimensional (2D) SDS-PAGE analysis, 1 × 10^9^ purified *E. cuniculi* spores were used for protein sample preparation following the procedures and buffers recommended in the ReadyPrep 2D-Starter Kit (Biorad; Hercules, CA) with slight modifications. The spore pellet was digested for 30 minutes at boiling temperature in denaturing buffer containing 0.05% SDS and 0.1% 2-mercaptoethanol. The supernatant was transferred to a new tube, and the free SDS was removed using the SDS-Out Reagent (Pierce/Thermo Scientific; Rockford, IL). The buffer was exchanged for the ReadyPrep2D Rehydration/Sample buffer using a Microcon (YM-10) Centrifugal Filter Device (Millipore; Billerica, MA). The buffer volume equivalent of 2.5 × 10^8^ spores was used to rehydrate two 11 cm pH 4–7 IPG strips according to the ReadyStrip IPG protocol (Biorad). The strips were focused using a Biorad Protean IEF cell and the standard recommendations for programming. For the second dimension, the gel strips were equilibrated in the kit Equilibration Buffer I and II and precast Criterion 8–16% Tris-HCl SDS-PAGE gels (Biorad) were used. For Western blots, the gels were transferred as described in [6]. 

To identify the Hsp70-related proteins, gel spots from Coomassie stained 2D-SDS-PAGE gels were excised and submitted for commercial protein identification by trypsin digestion and tandem mass spectrometry using nanoliquid chromatography/tandem mass spectrometry (Midwest Bio Services, Overland Park, KS). The acquired data were analyzed using Sequest database searching software.

## 3. Results

### 3.1. Cloning and Heterologous Expression of E. cuniculi Recombinant Hsp70-Related Protein

To identify an internal protein for comparison to spore surface proteins, we focused on heat-shock proteins (Hsp), which are usually located internally in the cytosol, endoplasmic reticulum, or mitochondria. Analysis of the *E. cuniculi* genome database revealed several candidate Hsp70-related genes, some of which have been characterized including the canonical mitochondrial organellar heat shock protein [7–9]. However, for our purposes, we selected a highly expressed Hsp70 family member from *E. cuniculi* (ECU02_0100; “C1”), which contains all three Hsp70 protein family signature motifs of conserved family members identified by Prosite database scanning [10]. In addition, this protein contains an N-terminal signal peptide allowing it to be translocated into the endoplasmic reticulum (ER) and a C-terminal ER targeting sequence (“REEL”), which would retain the protein in the ER.

**Figure 1:**
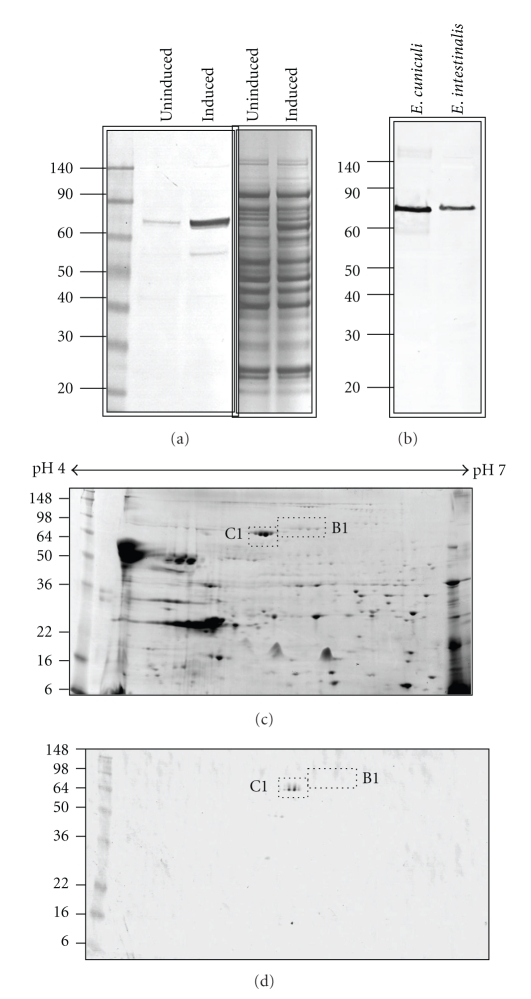
Heterologous expression of *E. cuniculi* C1 Hsp70 protein as a histidine fusion protein in *E. coli* and Western blot analysis using purified C1 antibodies. A Coomassie stained SDS-PAGE gel ((a), right panel) of uninduced and IPTG induced expression of the recombinant protein shows a ~76 kDa protein in the induced lane. A Western blot of the SDS-PAGE gel was performed using histidine-tag-specific antibodies ((a), left panel) confirmed the recombinant protein induction. (b) A single ~76 kDa band was detected on 1D Western blot of *E. cuniculi* and *E. intestinalis* total spore protein using purified C1 Hsp70 protein antibodies. (c) The C1 (ECU02_0100) and B1 (ECU03_0520) Hsp70-related proteins were identified from a Coomassie stained 2D SDS-PAGE gel of *E. cuniculi* total spore protein by MALDI-MS analysis of trypsin digested gel spots. (d) Western analysis of the 2D gel using the C1 specific antibodies detected only the C1 protein (d).

The open reading frame that encodes from the N-terminal methionine residue to the C-terminal leucine residue immediately prior to the termination codon of C1 was amplified, cloned, and expressed in *E. coli*. The expressed recombinant protein was similar to the predicted size of 76.2 kDa (Figure 1(a)) and was used to immunize naïve rabbits. In one-dimensional Western blot analysis using purified spore protein from both *E. intestinalis* and *E. cuniculi,* the anti-C1 Hsp70 protein antibodies recognized a single protein band from both species (Figure 1(b)). However, our analysis of the *E. cuniculi* genome database identified a second Hsp70-related protein with a predicted size of 74.8 kDa (ECU03_0520; “B1”). Because the masses of these two proteins are so similar and the predicted amino acid identity is 28.3%, the single band observed in 1D Westerns may represent recognition of both the C1 and B1 Hsps. Such reactivity would be difficult to distinguish using one-dimensional SDS-PAGE. Therefore, two-dimensional SDS-PAGE electrophoresis and Western blotting analysis were performed with purified *E. cuniculi* spore proteins and the anti-C1 Hsp70 antibodies. Matrix-assisted laser desorption ionization mass spectrometry analysis of trypsin digested Coomassie gel spots identified both the C1 and B1 Hsp70 proteins (Figure 1(c)). Peptide coverage represented 38 and 24% of the C1 and B1 proteins, respectively. Interestingly, the B1 protein, which is predicted to be 74.8 kDa, is slightly larger in mass on the 2D SDS-PAGE gel than the predicted 76.2 kDa C1 protein. It is possible that the removal of the signal peptide from the C1 could account for the size discrepancy. Nonetheless, Western blotting of the two dimensional SDS-PAGE shows that the anti-C1 Hsp70 antibodies are specific for the C1 Hsp70-related protein and do not cross react with the similar sized B1 protein (Figure 1(d)).

### 3.2. Immunolocalization of C1 Hsp70-Like Protein in E. cuniculi Spores

**Figure 2:**
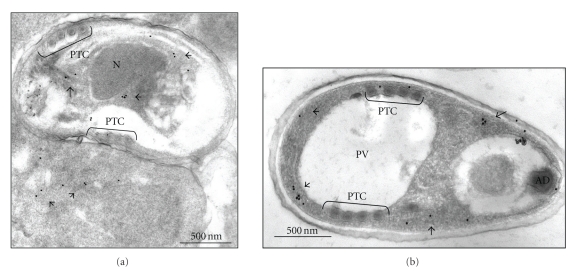
Immuno-TEM of *E. cuniculi* infected rabbit kidney cells. The infected cells were prepared according to the protocol described. Ultrathin Lowicryl resin sections were cut and reacted with C1 Hsp70 specific antibodies (1 : 10) and a secondary antibody conjugated to 15-nm gold particles (1 : 200). The developmental stages include immature meronts and sporonts (the lower and upper spores in (a), resp.) and a mature spore (b). Abbreviations are as follows: PTC, polar tube coil; N, nucleus; PV, posterior vacuole; AD, anchoring disk. Each bar indicates a 500-nm scale.

To confirm that the C1 Hsp70-like protein does not localize to the spore wall, immunogold labeled transmission electron microscopy (immuno-TEM) of *E. cuniculi* infected RK13 cells was performed using the purified anti-C1 Hsp70 antibodies. The antibodies recognize protein in both immature (Figure 2(a)), and mature spores (Figure 2(b)). The C1 Hsp70 protein does not appear to be located in one specific area, but rather is spread throughout the cytoplasm. In microsporidia, the membranous ER and associated ribosomes of spores are difficult to visualize, especially in immunolocalization TEM resin. However, in structural studies, the ER has been located both surrounding the nucleus and in other parts of the cytoplasm [11]. The immunolabeling of the C1 Hsp70 protein in Figure 2 appears to match the ER structural profile. No significant immunolabeling was evident on the outside surfaces of developing or mature spores or within host cells that did not contain parasitophorous vacuoles (data not shown).

### 3.3. Anti-C1 Antibodies Do Not Inhibit E. cuniculi Spore Adherence to Host Cells

**Figure 3:**
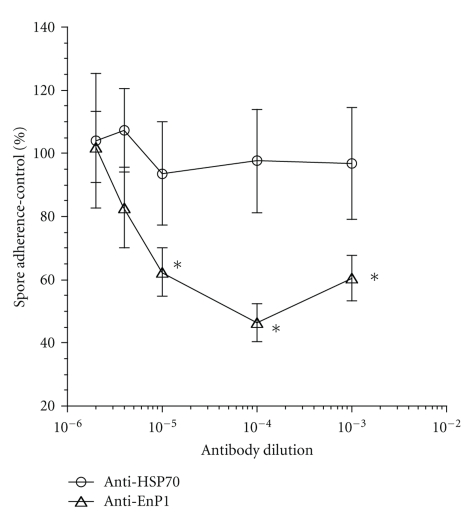
Purified antibodies specific for the internal C1 Hsp70 protein does not inhibit *E. cuniculi* spore adherence to rabbit kidney host cells. Increasing concentrations of C1 antibodies (open circles) or EnP1 specific antibodies (open triangle) were incubated with spores in the presence of confluent host cells. Control samples excluded antibodies. After 4-hours, unbound spores were removed by washing and the bound spores were quantified by immunofluorescence assay. Each antibody dilution sample was performed in triplicate. Statistical significance is indicated with asterisks (*P* < .0001).

The previous studies have shown that microsporidia infection of host cells may involve an initial attachment of spores to host cell surfaces, which precedes spore activation, germination, and polar filament extrusion [3, 6]. Spore attachment, and thus host cell infection, can be inhibited with the addition of either sulfated glycosaminoglycans, a common host cell surface glycan, or exogenous endospore protein-1 (EnP1). EnP1 is found in both the endospore and exospore regions of spore walls and is an adherence ligand potentially involved in the spore activation process [6, 12]. Addition of either recombinant EnP1 or anti-EnP1 antibodies to spore adherence assays significantly reduces the number of adherent spores. To confirm that the anti-C1 Hsp70 antibodies, which specifically recognize the nonsurface associated ER Hsp70 protein, do not affect spore attachment, spore/host cell adherence assays were performed using both the anti-C1 antibodies and the anti-EnP1 antibodies (Figure 3). Adding increasing amounts of anti-EnP1 antibodies leads to dose-dependant decreases in spore adherence, as was expected. However, equivalent quantities of anti-C1 antibodies have no affect on spore adherence to host cell surfaces, confirming that the internally located C1 Hsp70 is not involved in adherence.

## 4. Discussion

The previous studies involving Hsp70 proteins of microsporidia have revolved around phylogeny, in part due to the fact that microsporidia are “amitochondriate” organisms [7–9, 13]. During microsporidia genome sequencing efforts, a mitochondria-associated heat shock protein was identified, despite the fact that microsporidia have no identifiable mitochondria organelle. Studies involving this Hsp eventually identified an “ancient” mitochondria organelle called a mitosome [9, 14]. This discovery placed microsporidia in a small group of amitochondriates, which includes *Giardia intestinalis*, *Trichomonas vaginalis*, and *Entamoeba histolytica* [14, 15]. Further studies revealed that several iron-sulphur cluster assembly proteins were located in the mitosome based on their co-localization with the mitosome-associated Hsp70 family member [16]. 

Most cells have multiple members of the Hsp70 family of heat shock proteins that function in a variety of manners. The most recognized functions of Hsp70s are to assist in protein folding and trafficking and coping with protein denaturation in response to increased stress, such as heat [4]. Interestingly, studies have shown that within the cell, Hsp70 family members may also exert a pro-survival affect, combating the intrinsic apoptotic pathway activation in neuronal cells [17]. Hsp72 inhibits the release of cytochrome-c from the mitochondria, inhibits apoptosis inducing factor translocation to the nucleus, and interferes with recruitment of procaspase-9 into the apoptosome [18]. Recently, it has also been shown that some Hsp70 family members can be released from cells to serve as a physiological alarm signal for cell trauma or to act as a pro- or anti-inflammatory mediators [19–21]. Either through active transport or passive release due to cellular destruction, the released Hsp proteins exert their affect beyond the cell from which they originated. In our immuno-TEM analysis, we did not see significant labeling in any host cell organelle. Nor was there significant labeling in the extracellular spaces within the parasitophorous vacuole indicating that the ER associated Hsp70-like protein may not be secreted. It remains to be seen whether the *E. cuniculi* cytosolic Hsp70-related protein (ECU03_0520; B1) that does not contain an ER retention signal or a signal peptide is released from developing or mature spores.

The C1 Hsp70 protein contains an ER retention signal at the C-terminal end. Although the ER has not been extensively studied in microsporidia, structural studies have identified the ER as membranous elements arranged in parallel cisternae covered by ribosomes [11]. These elements both surround the nucleus and are dispersed throughout the cytoplasm. Both the spore-wall and polar-tube proteins have predicted signal peptides for ER translocation and trafficking [22–24]. And, recent studies indicate that microsporidia have the necessary machinery, albeit reduced and modified, for translocation of polypeptide chains into the ER [25]. Furthermore, Golgi complex studies in microsporidia suggest they do not create budding vesicles with typical eukaryotic coat proteins, but have instead tubular networks that directly connect to the ER, the polar tube membrane, and the plasma membrane [26]. Thus, the extensive network of membranes would be distributed throughout the spore cytoplasm. The varied immuno-EM cytosolic labeling of C1 Hsp70 protein shown here would be indicative of an extensive membranous ER/Golgi secretory pathway. 

In summary, we have cloned and expressed an ER associated member of the Hsp70 family from *E. cuniculi*. Antibodies generated against this protein specifically recognize C1 Hsp70 from microsporidia and localize it to internal structures of spores. Moreover, the anti-C1 antibodies did not inhibit spore adherence. As a result, the C1 Hsp70 protein and the antibody are important tools for research in the field of microsporidia and will be used as internal markers for comparisons during our studies on spore adherence.
